# Prescription Drug Use Among US Adults

**DOI:** 10.1001/jamanetworkopen.2026.33929

**Published:** 2026-09-15

**Authors:** Maria J. Monroy-Iglesias, Peng Wang, Yashasvini Sampathkumar, Kelli O’Connell, Andrew T. Chan, Mengmeng Du, Jennifer S. Haas, Edward L. Giovannucci, Elizabeth D. Kantor

**Affiliations:** 1 Department of Epidemiology and Biostatistics, Memorial Sloan Kettering Cancer Center, New York, New York; 2 Channing Division of Network Medicine, Brigham and Women’s Hospital, Boston, Massachusetts; 3 Division of Gastroenterology, Massachusetts General Hospital, Boston; 4 Clinical and Translational Epidemiology Unit, Massachusetts General Hospital, Boston; 5 Division of General Medicine, Massachusetts General Hospital, Boston; 6 Departments of Nutrition and Epidemiology, Harvard T.H. Chan School of Public Health, Boston, Massachusetts

## Abstract

This cross-sectional study evaluates prescription drug use, polypharmacy, and therapeutic drug class use reported by participants in the National Health and Nutrition Examination Survey from 1999 to 2020.

## Introduction

Prescription drug use in the US has steadily increased, likely reflecting changes in chronic disease burden, clinical practice, and medication availability.^1,2,3,4^ Nationally representative data have documented prescription medication use and polypharmacy, including a National Health and Nutrition Examination Survey (NHANES) trend analysis (1999-2012),^1^ cross-sectional national estimates,^3,4^ and studies on specific populations or drug classes.^2,5^ Accordingly, we examined prescription drug use, polypharmacy, and therapeutic drug class use to provide an updated assessment of long-term drug use patterns across the US adult population.

## Methods

We analyzed adults 20 years or older participating in NHANES from January 1999 through March 2020, extending an analysis of prescription drug use trends covering 1999 through 2012.^1^ The January 1999 to December 2000 cycle marked the start of continuous NHANES data collection; the combined nationally representative January 2017 to March 2020 cycle was the final cycle to include detailed prescription drug data. Participants with missing information on prescription drug use were excluded. All NHANES participants provided written informed consent. In accordance with 45 CFR §46.104, this cross-sectional study was exempt from ethics review because publicly available data were used. We followed the STROBE reporting guideline.

Prescription drug use in the previous 30 days was ascertained during household interviews; participants were asked to provide medication containers or to report medication names when containers were unavailable. Reported drugs were linked to the Lexicon Plus database (Cerner Multum). Combination products were analyzed at the active ingredient level rather than as individual products. Topical and other locally administered medications (eg, ophthalmic, otic, rectal, vaginal preparations) were excluded.

Survey-weighted estimates calculated the prevalence of overall prescription drug use, polypharmacy (≥5 prescription drugs), and therapeutic drug class use across survey cycles. Overall and polypharmacy estimates were stratified by age (20-39, 40-64, ≥65 years), sex, and self-reported race and ethnicity (Mexican American, non-Hispanic Black, and non-Hispanic White; other groups excluded due to insufficient sample sizes) to assess subgroup differences. Survey-weighted binomial regression models were used to estimate prevalence ratios and test for linear trends over time. Absolute differences in prevalence between the first (January 1999 to December 2000) and last (January 2017 to March 2020) survey cycles were also calculated. Analyses were conducted from March to July 2025 using R 4.3.2 (R Project for Statistical Computing).

## Results

Among 58 201 adults (mean [SD] age, 47.0 [17.1] years; 52.0% [weighted] females) included in the analysis, 8.1% self-identified as Mexican American, 11.3% as non-Hispanic Black, and 67.6% as non-Hispanic White. Prescription drug use increased from 46.7% in January 1999 to December 2000 to 56.4% in January 2017 to March 2020, while polypharmacy increased from 7.8% to 16.7% within those periods (Figure). Increases in prescription drug use and polypharmacy were observed across age, sex, and racial and ethnic groups. The largest increase in polypharmacy occurred among adults 65 years or older (21.9% to 40.7%), whereas polypharmacy remained uncommon among adults aged 20 to 39 years (1.3% to 3.4%) (Figure).

**Figure.**
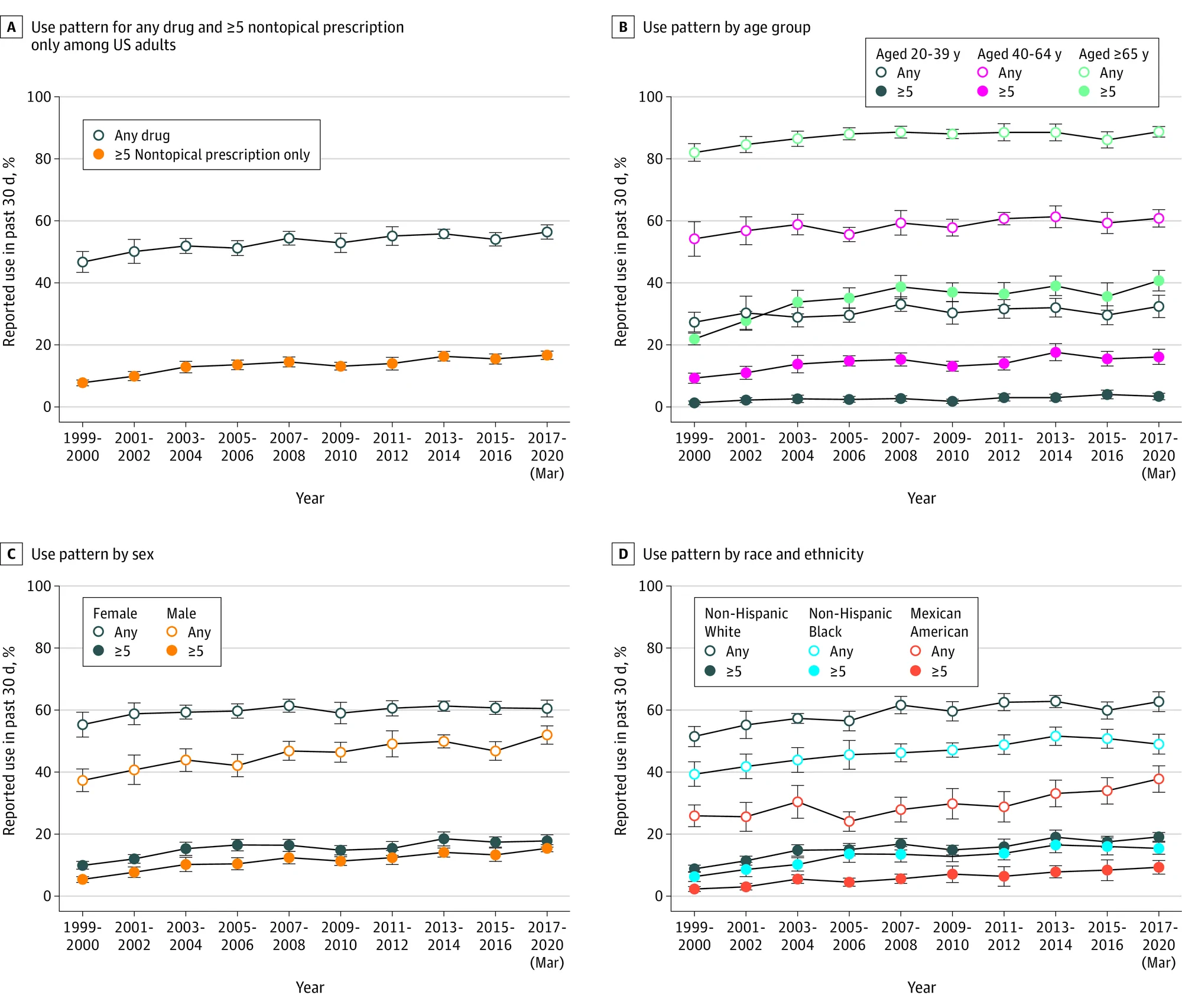
Line Graphs of Trends in Nontopical Prescription–Only Drug Use in Past 30 Days, Overall, and by Participant Group From January 1999 to March 2020 *P* for trend <.001 for all. All data were weighted to be nationally representative. Error bars represent 95% CIs.

The largest increases in drug use from first to last cycles were observed for antihypertensives (18.3% [95% CI, 16.4%-20.2%] to 30.0% [95% CI, 28.4%-31.6%]), antihyperlipidemics (7.5% [95% CI, 6.8%-8.3%] to 20.3% [95% CI, 18.3%-22.4%]), antidiabetics (4.6% [95% CI, 3.8%-5.4%] to 11.1% [95% CI, 10.3%-11.8%]), central nervous system stimulants (14.0% [95% CI, 12.3%-15.7%] to 20.2% [95% CI, 18.9%-21.6%]), and psychotherapeutics (7.3% [95% CI, 6.1%-8.4%] to 14.7% [95% CI, 13.3%-16.1%]) (Table). Within these therapeutic classes, use of renin-angiotensin system agents, anticonvulsants, and antidepressants increased substantially. Use of anticoagulants and antiplatelets, primarily reflecting anticoagulants, more than doubled (2.0% [95% CI, 1.5%-2.4%] to 4.7% [95% CI, 4.0%-5.3%]).

**Table.** Prevalence of Nontopical Prescription–Only Drug Use in the Past 30 Days Among NHANES Participants, January 1999 to March 2020^a^

Variable	**Weighted prevalence, % (95% CI)**				Prevalence ratio (95% CI)	*P* value for trend
	NHANES 1999-2000 (n = 4813)	NHANES 2011-2012 (n = 5517)	NHANES 2017-2020 (n = 9101)	Prevalence difference		
Any nontopical prescription–only drugs	46.7 (43.4 to 50.1)	55.1 (52.1 to 58.1)	56.4 (54.1 to 58.7)	9.6 (5.5 to 13.8)	1.2 (1.1 to 1.3)	<.001
≥2 Nontopical prescription–only drugs	30.7 (28.4 to 32.9)	39.2 (36.0 to 42.4)	43.3 (41.2 to 45.5)	12.7 (9.5 to 15.8)	1.4 (1.3 to 1.5)	<.001
≥5 Nontopical prescription–only drugs	7.8 (6.8 to 8.7)	14.0 (11.9 to 16.0)	16.7 (15.3 to 18)	8.9 (7.2 to 10.6)	2.1 (1.8 to 2.5)	<.001
Antihypertensives	18.3 (16.4 to 20.2)	27.0 (24.1 to 29.8)	30.0 (28.4 to 31.6)	11.7 (9.2 to 14.2)	1.6 (1.5 to 1.8)	<.001
β Blockers	5.5 (4.7 to 6.3)	9.8 (8.0 to 11.7)	11.5 (10.3 to 12.7)	6.0 (4.6 to 7.4)	2.1 (1.8 to 2.5)	<.001
Calcium channel blockers	3.7 (3.1 to 4.2)	5.6 (4.6 to 6.6)	6.6 (6.0 to 7.3)	2.9 (2.1 to 3.8)	1.8 (1.5 to 2.1)	<.001
RAS-acting agents	8.5 (7.3 to 9.7)	17.3 (15 to 19.5)	19.6 (18.2 to 20.9)	11.1 (9.3 to 12.9)	2.3 (2.0 to 2.7)	<.001
Diuretics	8.7 (7.2 to 10.2)	12.4 (10.9 to 13.9)	11.1 (10.0 to 12.1)	2.4 (0.5 to 4.2)	1.3 (1.0 to 1.5)	.002
α Blockers	1.6 (1.2 to 1.9)	2.1 (1.6 to 2.7)	2.9 (2.4 to 3.4)	1.3 (0.8 to 1.9)	1.9 (1.4 to 2.4)	<.001
Central α agonists	0.8 (0.5 to 1.1)	1.0 (0.7 to 1.3)	0.8 (0.5 to 1.1)	0 (−0.4 to 0.4)	1.0 (0.6 to 1.8)	.40
Antiarrhythmics	5.0 (4.6 to 5.4)	2.8 (2.1 to 3.5)	2.8 (2.1 to 3.4)	−2.2 (−3.0 to −1.4)	0.6 (0.4 to 0.7)	<.001
Antianginals	1.5 (1.1 to 2.0)	1.0 (0.8 to 1.2)	0.9 (0.6 to 1.1)	−0.7 (−1.2 to −0.2)	0.6 (0.4 to 0.8)	<.001
Vasodilators	0.2 (0.1 to 0.3)	0.5 (0.3 to 0.6)	0.5 (0.4 to 0.7)	0.3 (0.1 to 0.5)	2.6 (1.5 to 4.4)	<.001
Antihyperlipidemics	7.5 (6.8 to 8.3)	18.4 (16.2 to 20.6)	20.3 (18.3 to 22.4)	12.8 (10.6 to 15.0)	2.7 (2.3 to 3.1)	<.001
Antidiabetics	4.6 (3.8 to 5.4)	8.2 (7.2 to 9.3)	11.1 (10.3 to 11.8)	6.4 (5.3 to 7.6)	2.4 (2.0 to 2.9)	<.001
Bone resorption inhibitors	0.6 (0.3 to 0.8)	0.8 (0.5 to 1.1)	1.0 (0.7 to 1.3)	0.4 (0 to 0.8)	1.7 (1.0 to 2.9)	.08
Antigout agents	0.7 (0.5 to 0.9)	1.1 (0.7 to 1.5)	1.5 (1.2 to 1.9)	0.9 (0.4 to 1.3)	2.3 (1.5 to 3.4)	<.001
CNS agents	14.0 (12.3 to 15.7)	17.7 (15.3 to 20.2)	20.2 (18.9 to 21.6)	6.2 (4.0 to 8.4)	1.4 (1.3 to 1.7)	<.001
Analgesics	8.6 (7.4 to 9.7)	9.1 (7.0 to 11.1)	9.1 (8.0 to 10.3)	0.5 (−1.1 to 2.2)	1.1 (0.9 to 1.3)	.04
Anticonvulsants	2.1 (1.6 to 2.5)	5.4 (4.5 to 6.4)	8.5 (7.4 to 9.6)	6.4 (5.2 to 7.6)	4.1 (3.2 to 5.3)	<.001
Anxiolytics, sedatives, or hypnotics	3.5 (2.5 to 4.4)	4.2 (3.3 to 5.2)	4.3 (3.8 to 4.9)	0.9 (−0.2 to 2.0)	1.3 (0.9 to 1.7)	.002
Muscle relaxants	1.2 (0.9 to 1.6)	2.5 (1.7 to 3.4)	2.6 (2.3 to 3.0)	1.4 (0.8 to 1.9)	2.1 (1.5 to 2.9)	<.001
Antiemetic or antivertigo agents	1.2 (0.7 to 1.7)	1.2 (0.9 to 1.6)	1.6 (1.1 to 2.0)	0.4 (−0.3 to 1.0)	1.3 (0.8 to 2.1)	.60
Antiparkinson agents	0.5 (0.2 to 0.7)	1.2 (0.7 to 1.6)	1.1 (0.7 to 1.4)	0.6 (0.2 to 1.0)	2.2 (1.2 to 4.2)	<.001
CNS stimulants	NR^b^	1.8 (1.0 to 2.6)	1.8 (1.2 to 2.5)	1.4 (0.6 to 2.1)	4.2 (1.8 to 9.6)	<.001
Antidementia agents	NR^b^	0.6 (0.4 to 0.8)	0.8 (0.5 to 1.0)	0.6 (0.3 to 0.9)	4.8 (2.0 to 11.6)	<.001
Hormones or hormone modifiers	16.3 (14.9 to 17.7)	13.2 (11.6 to 14.8)	15.0 (13.8 to 16.2)	−1.3 (−3.1 to 0.6)	0.9 (0.8 to 1.0)	.02
Sex hormones	9.8 (8.6 to 11.0)	5.3 (4.2 to 6.4)	4.5 (3.7 to 5.2)	−5.3 (−6.8 to −3.9)	0.5 (0.4 to 0.6)	<.001
Corticosteroids	2.8 (2.2 to 3.3)	1.6 (1.1 to 2.0)	1.9 (1.5 to 2.3)	−0.9 (−1.5 to −0.2)	0.7 (0.5 to 0.9)	.002
Thyroid hormones	5.2 (4.4 to 5.9)	6.4 (5.2 to 7.6)	8.4 (7.5 to 9.2)	3.2 (2.1 to 4.3)	1.6 (1.4 to 1.9)	<.001
5-ARIs	NR^b^	1.0 (0.7 to 1.3)	1.3 (0.8 to 1.7)	1.1 (0.6 to 1.6)	7.2 (2.8 to 18.7)	<.001
Psychotherapeutic agents	7.3 (6.1 to 8.4)	13.6 (11.5 to 15.7)	14.7 (13.3 to 16.1)	7.4 (5.6 to 9.3)	2.0 (1.7 to 2.4)	<.001
Antidepressants	6.8 (5.8 to 7.9)	12.9 (10.6 to 15.1)	14.2 (12.7 to 15.6)	7.3 (5.6 to 9.1)	2.1 (1.7 to 2.5)	<.001
Antipsychotics	0.9 (0.5 to 1.4)	1.4 (1.0 to 1.8)	1.7 (1.2 to 2.2)	0.7 (0.1 to 1.4)	1.8 (1.0 to 3.2)	.002
Respiratory agents	4.3 (3.7 to 5.0)	6.1 (4.7 to 7.5)	5.3 (4.6 to 6.1)	1.0 (0 to 2.0)	1.2 (1.0 to 1.5)	.005
Bronchodilators	3.2 (2.5 to 3.9)	5.2 (3.9 to 6.4)	3.4 (2.7 to 4.2)	0.2 (−0.9 to 1.3)	1.1 (0.8 to 1.5)	.10
Inhalants	1.8 (1.4 to 2.2)	2.3 (1.5 to 3.1)	2.0 (1.4 to 2.7)	0.2 (−0.5 to 1.0)	1.1 (0.8 to 1.7)	.10
Leukotriene modifiers	0.4 (0.3 to 0.5)	1.1 (0.6 to 1.6)	1.9 (1.4 to 2.5)	1.5 (1.0 to 2.1)	4.6 (3.1 to 7.0)	<.001
Antihistamines	NR^b^	NR^b^	NR^b^	0 (−0.1 to 0)	0.6 (0.1 to 2.5)	.01
Anti-infectives^c^	6.3 (5.6 to 7.0)	5.5 (4.8 to 6.2)	5.4 (4.8 to 6.0)	−0.9 (−1.8 to 0.1)	0.9 (0.7 to 1.0)	.01
Antibiotics	5.0 (4.4 to 5.7)	3.5 (3.0 to 4.1)	3.7 (3.1 to 4.3)	−1.4 (−2.3 to −0.4)	0.7 (0.6 to 0.9)	<.001
Antivirals	NR^b^	1.4 (0.9 to 1.8)	1.2 (0.8 to 1.5)	0.6 (0.1 to 1.2)	2.3 (1.1 to 4.7)	<.001
Urinary anti-infectives	0.7 (0.4 to 0.9)	0.6 (0.3 to 0.9)	0.7 (0.3 to 1.0)	0 (−0.4 to 0.4)	1 (0.5 to 1.9)	.60
Antimalarials	NR^b^	0.3 (0.2 to 0.4)	0.3 (0.2 to 0.4)	0 (−0.2 to 0.2)	1 (0.5 to 2.3)	.10
Antifungals	NR^b^	0.2 (0.1 to 0.3)	NR^b^	0.1 (−0.2 to 0.5)	1.5 (0.5 to 4.5)	.50
Coagulation modifiers	2.0 (1.5 to 2.4)	3.5 (3.0 to 4.1)	4.7 (4.0 to 5.3)	2.7 (2.0 to 3.5)	2.4 (1.9 to 3.1)	<.001
Anticoagulants	1.3 (0.9 to 1.7)	1.7 (1.3 to 2.1)	2.3 (1.9 to 2.8)	1.1 (0.5 to 1.7)	1.8 (1.3 to 2.7)	<.001
Antiplatelets	0.6 (0.4 to 0.7)	1.9 (1.4 to 2.3)	2.4 (2.0 to 2.8)	1.8 (1.4 to 2.3)	4.3 (3.1 to 6.0)	<.001
GI agents	1.6 (1.2 to 2.1)	2.4 (1.7 to 3.1)	3.9 (3.3 to 4.4)	2.2 (1.5 to 3.0)	2.4 (1.7 to 3.3)	.01
Antineoplastics	1.1 (0.8,1.5)	0.8 (0.4 to 1.1)	1.2 (0.9 to 1.5)	0.1 (0.4 to 0.6)	1.1 (0.7 to 1.7)	.56
GU tract agents	1.0 (0.4 to 1.5)	1.1 (0.7 to 1.5)	1.5 (1.3 to 1.8)	0.6 (0 to 1.2)	1.6 (0.9 to 3.0)	.01
Antirheumatics	NR^b^	NR^b^	0.4 (0.3 to 0.6)	0.3 (0.1 to 0.5)	4.2 (1.1 to 16.0)	.01

Abbreviations: 5-ARIs, 5α-reductase inhibitors; CNS, central nervous system; GI, gastrointestinal; GU, genitourinary; NHANES, National Health and Nutrition Examination Survey; NR, not reported; RAS, renin angiotensin system.

^a^ This analysis was based on the NHANES prescription medication inventory and therefore does not comprehensively capture over-the-counter medication use.

^b^ Data withheld due to relative SE being higher than 30%; results for a given survey cycle were not presented if the relative SE ([SE of prevalence/prevalence] × 100) was higher than 30%, consistent with NHANES analytic guidelines. Drugs with 70% or more withholding cycles were not shown.

^c^ Estimates for anti-infective agents should be interpreted cautiously because these medications are often used short term and may be sensitive to the timing of data collection, despite continuous NHANES data collection throughout the year.

Hormone and hormone modifier use remained relatively stable overall (16.3% [95% CI, 14.9%-17.7%] to 15.0% [95% CI, 13.8%-16.2%]), although sex hormone use declined (9.8% [95% CI, 8.6%-11.0%] to 4.5% [95% CI, 3.7%-5.2%]) while thyroid hormone use increased (5.2% [95% CI, 4.4%-5.9%] to 8.4% [95% CI, 7.5%-9.2%]). Decreases were observed for antiarrhythmics (5.0% [95% CI, 4.6%-5.4%] to 2.8% [95% CI, 2.1%-3.4%]) and anti-infectives (6.3% [95% CI, 5.6%-7.0%] to 5.4% [95% CI, 4.8%-6.0%]) (Table).

## Discussion

In this nationally representative study, prescription drug use increased between January 1999 and March 2020, with 56.4% of adults reporting at least 1 prescription drug use by the final survey cycle. Polypharmacy also increased substantially, particularly among adults 65 years or older. The largest increases occurred in use of cardiometabolic disease drugs.

These findings extend the 1999 to 2012 NHANES analysis^1^ and suggest that long-term increases in prescription drug use and polypharmacy have continued since then. Although direct comparisons should be made cautiously because of small differences in analytical approach, overall patterns in the present study were consistent with those in the previous analysis and other nationally representative reports of prescription drug use.^1^ These upward trends persisted through the final NHANES cycle including prescription drug data. While factors underlying such usage were not identified, the increasing use of cardiometabolic disease drugs was consistent with evolving clinical guidelines, expanded therapeutic options, and increasing burden of chronic disease in the US.^6,7,8,9,10^

A study limitation is that over-the-counter medication data were not comprehensively captured, resulting in underestimation of overall drug use. Nevertheless, this analysis provides a benchmark for long-term prescribing patterns. Because NHANES no longer collects prescription drug data, continued national surveillance is needed to understand evolving drug use patterns and to inform clinical practice.

## Notes

Supplement.
Data Sharing Statement
