# Supplementary material for: Prescription Drug Use Among US Adults
Source: JAMA Netw Open. 2026 Sep 15;9(9):e2633929. doi: 10.1001/jamanetworkopen.2026.33929 (PMC13579433; doi:10.1001/jamanetworkopen.2026.33929)
Supplement: Supplementary file 1 [file jamanetwopen-e2633929-s001.pdf]

Data Sharing Statement

Monroy-Iglesias. Prescription Drug Use Among US Adults. *JAMA Netw Open*. Published September 15, 2026. doi:10.1001/jamanetworkopen.2026.33929

Data
Data available: Yes
Data types: Deidentified participant data
How to access data: NHANES data is publicly available to download from their website.
When available: With publication

Supporting Documents
Document types: None

Additional Information
Who can access the data: NHANES data is publicly available to download from their website.
Types of analyses: Any purpose.
Mechanisms of data availability: NHANES data is publicly available to download from their website.
